# Insulin adherence behaviours and barriers in the multinational Global Attitudes of Patients and Physicians in Insulin Therapy study

**DOI:** 10.1111/j.1464-5491.2012.03605.x

**Published:** 2012-05

**Authors:** M Peyrot, A H Barnett, L F Meneghini, P-M Schumm-Draeger

**Affiliations:** 1 Department of Sociology, Loyola University of MarylandBaltimore, MD, USA; 2 Department of Medicine, Johns Hopkins UniversityBaltimore, MD, USA; 3 University of Birmingham and BioMedical Research Unit, Heart of England NHS Foundation TrustBirmingham, UK; 4 Department of Medicine, University of MiamiMiami, FL, USA; 5 Department of Medicine, Bogenhausen Academic Teaching HospitalMunich, Germany

**Keywords:** adherence, insulin therapy, psychosocial, questionnaire

## Abstract

**Aims:**

To examine patient and physician beliefs regarding insulin therapy and the degree to which patients adhere to their insulin regimens.

**Methods:**

Internet survey of 1250 physicians (600 specialists, 650 primary care physicians) who treat patients with diabetes and telephone survey of 1530 insulin-treated patients (180 with Type 1 diabetes, 1350 with Type 2 diabetes) in China, France, Japan, Germany, Spain, Turkey, the UK or the USA.

**Results:**

One third (33.2%) of patients reported insulin omission/non-adherence at least 1 day in the last month, with an average of 3.3 days. Three quarters (72.5%) of physicians report that their typical patient does not take their insulin as prescribed, with a mean of 4.3 days per month of basal insulin omission/non-adherence and 5.7 days per month of prandial insulin omission/non-adherence. Patients and providers indicated the same five most common reasons for insulin omission/non-adherence: too busy; travelling; skipped meals; stress/emotional problems; public embarrassment. Physicians reported low patient success at initiating insulin in a timely fashion and adjusting insulin doses. Most physicians report that many insulin-treated patients do not have adequate glucose control (87.6%) and that they would treat more aggressively if not for concern about hypoglycaemia (75.5%). Although a majority of patients (and physicians) regard insulin treatment as restrictive, more patients see insulin treatment as having positive than negative impacts on their lives.

**Conclusions:**

Glucose control is inadequate among insulin-treated patients, in part attributable to insulin omission/non-adherence and lack of dose adjustment. There is a need for insulin regimens that are less restrictive and burdensome with lower risk of hypoglycaemia.

## Introduction

While patients with Type 1 diabetes must take insulin to survive, many patients with Type 2 diabetes also require insulin supplementation in order to control persistent hyperglycaemia. However, patients with Type 2 diabetes often do not receive insulin or do not receive insulin in a timely manner [1–3]. Delays in insulin initiation and consequent exposure to prolonged periods of poor glycaemic control are believed to increase the severity and progression of macrovascular, as well as microvascular complications [4]. The American Diabetes Association and the European Association for the Study of Diabetes recently issued guidelines for the treatment of Type 2 diabetes that identified insulin as the most effective glucose-lowering agent and insulin replacement therapy as a key component of effective diabetes management over the course of the disease [5]. Moreover, among those receiving insulin therapy, regimen adherence, persistence and intensity may be poor [6–8], resulting in worse glucose control and increased hospital admissions for diabetes complications [9, 10].

Effective insulin therapy includes four critical accomplishments: initiation, adherence, persistence, intensification [11]. Each of these requires the involvement of both patient and healthcare provider: (1) providers must recommend/prescribe insulin and patients must fill their prescriptions and begin taking the medication; (2) providers must formulate an insulin regimen that patients can implement and patients must adhere to that regimen; (3) providers must renew prescriptions and patients must continue to refill and use their prescriptions; (4) providers must intensify when appropriate (increase dose and frequency of administration) and patients must accept and implement the intensified regimen. Unfortunately, there are failures at each juncture, some of which can be attributed to patients, some to providers and all, in part, because of the nature of the insulins and delivery systems that are available to patients and physicians.

There are a number of provider barriers to initiation of insulin therapy. Some of these relate to beliefs about the medication itself; some physicians believe that insulin therapy may not be effective, may result in weight gain, increase the risk of hypoglycaemia and have other side effects [12]. Physicians also may believe that insulin therapy is inconvenient and painful for patients and will result in patient dissatisfaction [13–15]. Especially important is the therapeutic orientation of the provider; i.e. whether or not the provider emphasizes normalization of blood glucose and modifies treatment to achieve glucose control targets [12]. Some barriers may be a function of the provider’s level of specialization and treatment experience [12, 16].

Patient barriers to some degree parallel those of providers; for example, concerns about efficacy, safety and weight gain [8, 15, 17]. But patients are also concerned about convenience, interference with daily living and social stigma [8, 11, 18]. While these concerns may be valid, patients also have inaccurate beliefs about insulin therapy, including the idea that insulin causes late-stage diabetes complications and is an indication of imminent deterioration and death, or is a result of patients’ failure to take good care of themselves [12]. Other barriers may be practical, including medication cost and difficulty with access [19].

This paper reports the results of the Global Attitudes of Patients and Physicians in Insulin Therapy study, a multinational survey of patients and providers regarding insulin therapy. This study examines patient and physician reports of insulin omission/non-adherence and the reasons for these events, physician perceptions of patient success with insulin treatment tasks, and patient and physician perceptions and beliefs about insulin therapy. The study allows a comparison of patient and physician responses for a number of beliefs and behaviours, which is important because prior research has suggested that there may be differences in beliefs and perceptions and these differences may interfere with optimal diabetes treatment [20, 21]. The study also permits a comparison of specialist and primary care physicians [12,19].

## Participants and methods

### Study design

The study consisted of cross-sectional surveys of patients and physicians in eight developed and developing countries: China, France, Japan, Germany, Spain, Turkey, the UK and the USA. The physician survey was conducted via the Internet and the patient survey was conducted through computer-assisted telephone interviewing. Each survey used one questionnaire that was translated into the primary language of each country. The questionnaires were developed with collaboration of Edelman, StrategyOne, Novo Nordisk and the authors. Before conducting the main survey phase, a pretest was conducted among primary care physicians and patients to check that the questionnaire was effective and unbiased and that there were no obvious errors or omissions. The final questionnaires were revised based upon the feedback obtained from the pretest.

The target physician sample size was set at 1250. Quotas were defined for the number of physicians—a minimum of 50 primary care physicians (internal medicine, general medicine and family practice) and 50 specialists (diabetologists and endocrinologists) in each country, with higher quotas in the USA (200 primary care physicians, 150 specialists), China (100 primary care physicians, 150 specialists) and the UK (100 primary care physicians, 50 specialists). Respondents were recruited via validated healthcare professional panels maintained by WorldOne Healthcare Research. Physician eligibility criteria were: in practice for more than 1 year since completing residency, see a minimum number of patients with diabetes per week (primary care physicians 5, specialists 10) and initiate insulin treatment for patients with diabetes.

The target patient sample size was set at 1500. Quotas were defined for the number of patients with Type 2 diabetes—a minimum of 135 in each country, with higher quotas in the USA (315), China (180) and the UK (180). Patients with Type 1 diabetes were captured during enrollment of those with Type 2 diabetes in whatever numbers were obtained. Respondents were recruited via panels of research consumers maintained by WorldOne Healthcare Research. Patient eligibility criteria were: age 18 years or older; use insulin to control blood sugar; Type 1 or Type 2 diabetes.

### Measures

The questionnaires assessed patient and physician reports of: frequency of insulin omission/non-adherence and reasons for these events; dissatisfaction with insulin therapy; perceptions of patient difficulties; and opinions of insulin therapy. The physician questionnaire also assessed perceptions of patient success with various insulin treatment tasks. The patient questionnaire also assessed perceptions of the impact of insulin treatment on their lives.

The key measure was insulin omission/non-adherence. For patients, this concept was assessed by a single item that assessed whether the respondents ever miss an insulin dose or do not take insulin exactly as prescribed and, if so, how many days this had happened in the last month. For patients, the frequency of insulin omission/non-adherence was calculated two ways: (1) based on the responses of all patients (treating those who said no to the original item as having zero days of insulin omission/non-adherence) and (2) the number of days reported by those who answered yes to the original item. For physicians, this concept was assessed by two questions; the first an item that assessed whether any of the respondents’ typical patients fail to take their insulin as prescribed. Physicians who responded yes were asked how many days in the last month a typical patient would miss an insulin dose or not take insulin exactly as prescribed for (1) basal insulin and (2) meal-related insulin. For physicians, the frequency of insulin omission/non-adherence was calculated two ways: (1) based on the responses of all physicians (treating those who said no to the original item as reporting zero days of insulin omission/non-adherence) and (2) the number of days reported by those who answered yes to the original item.

### Statistical analysis

Significance of differences between patient and physicians, between patients with Type 1 and Type 2 diabetes, and between specialists and primary care providers are analysed using the χ^2^ statistic. Other than data regarding characteristics of the study populations (Table 1), all data are weighted so that every country is equally represented. For the physician data, specialists and primary care physicians are weighted so that they are equally represented. For patients, data are weighted so that the percentage of Type 1 and Type 2 diabetes is the same for each country as for the overall study population. Weights maintain original sample sizes. Thus, the results are not influenced by disproportionate country sample sizes.

**Table 1:** Characteristics of study populations

Characteristic	Response

Patient sample	*n* = 1530
Country	
China	13.1%
France	10.5%
Germany	9.9%
Japan	9.8%
Spain	10.3%
Turkey	10.1%
UK	13.4%
USA	22.9%
Female	50.4%
Age (years, mean ± sd)	60.1 ± 13.7
Race/ethnicity	
White	44.2%
Asian	23.9%
Middle Eastern	9.4%
Hispanic	5.7%
Black	4.5%
None of the above	12.4%
Type 2 diabetes	88.2%
Duration of diabetes (years, mean ± sd)	14.7 ± 10.2
Duration of insulin treatment (years, mean ± sd)	8.6 ± 8.3
Insulin delivery system	
Pen only	74.2%
Syringe only	20.6%
Pen and syringe	3.7%
Other	1.5%
Physician sample	*n* = 1250
Country	
China	20.0%
France	8.0%
Germany	8.0%
Japan	8.0%
Spain	8.0%
Turkey	8.0%
UK	12.0%
USA	28.0%
Male	70.0%
Specialty	
Diabetology	17.2%
Endocrinology	30.8%
Family practice	16.1%
General practice	20.4%
Internal medicine	15.5%
Primary clinical setting	
Private practice office	42.4%
Hospital outpatient	31.7%
Hospital inpatient	10.4%
Community health centre	9.1%
Public health service	5.4%
Other	1.0%
Duration of clinical practice (years, mean ± sd)	17.0 ± 8.3
Type 1 patients (no. weekly, mean ± sd)	10.3 ± 13.5
Type 2 patients (no. weekly, mean ± sd)	56.7 ± 54.5
Type 2 patients using insulin (no. weekly, mean ± sd)	24.0 ± 28.2

### Ethical approval

The study, which received ethical approval from the Human Subjects Committee at Loyola University Maryland, complies with the recommendations of the 1964 Declaration of Helsinki and all relevant ethical standards.

## Results

### Study populations

Patient recruitment targets were met or exceeded in each country, resulting in a total sample of 1530 respondents (Table 1). Respondents were almost equally divided by gender, with a mean age of approximately 60 years (Type 1 diabetes ∼47 years, Type 2 diabetes ∼62 years). White people were the largest racial/ethnic group, but less than half the total sample. Most respondents had Type 2 diabetes and the mean duration of diabetes was almost 15 years (Type 1 diabetes ∼18 years, Type 2 diabetes ∼14 years). Respondents had been using insulin for an average of almost 9 years (Type 1 diabetes ∼15 years, Type 2 ∼8 years) and the majority used an insulin pen all or part of the time (Type 1 diabetes ∼76%, Type 2 diabetes ∼78%).

Physician recruitment targets were met in each country, resulting in a total sample of 1250 respondents (Table 1). Specialists (diabetologists and endocrinologists) made up almost half of the sample and primary care physicians were relatively equally divided among internal medicine, general medicine and family practice. Private offices and hospitals were the predominant practice sites. Respondents had been practicing for an average of 17 years and saw an average of over 30 insulin-treated patients during a week.

### Survey responses

**FIGURE 1:**
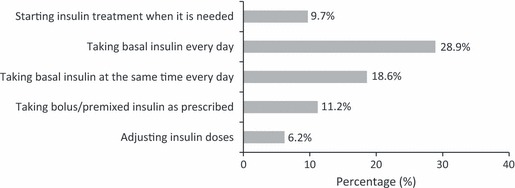
Physician report: patient success with insulin treatment tasks (*n* = 1250)*†. *Percentage of physicians reporting that their patients are very successful (vs. somewhat successful, not very successful, not at all successful, ‘don’t know’). †Specialists and primary care physicians not significantly different for any measure

Physicians rated patient success with insulin treatment tasks as low (Fig. 1). Only a minority of patients were rated as ‘very successful’ with any of the tasks, with very low rates for starting insulin when needed and adjusting doses. Less than one third of physicians rated patients as very successful in being able to take their basal insulin everyday (28.9%) or take their bolus/premixed insulin as prescribed (11.2%).

Patients and physicians reported a high level of insulin omission/non-adherence. One third of patients (33.2%) reported insulin omission/non-adherence, with a mean of 3.3 days in the last month; the calculated rate of insulin omission/non-adherence for the entire sample was 1.1 days. The majority of physicians (72.5%) reported that some of their typical patients do not take their insulin as prescribed (this percentage cannot be compared with the percentage of patients reporting insulin omission/non-adherence because physicians did not report the number or percentage of their patients who did so). For physicians who said yes, the reported days per month of insulin omission/non-adherence was 4.3 for basal insulin and 5.7 for meal-related insulin. The calculated rate of insulin omission/non-adherence for the entire sample was 3.1 days for basal insulin and 4.1 days for meal-related insulin. These data support the physicians’ report that there is less success with administration of bolus/premixed insulin than basal insulin (Fig. 1).

Patients and physicians were asked to choose the top three of 10 possible reasons for insulin omission/non-adherence (Table 2); one reason (whether omission/non-adherence was a result of forgetting) was volunteered by a substantial number of respondents. The absolute percentages reported for physicians cannot be compared with percentage of patients who report these reasons, because physicians report whether this is a reason for the behaviour of their ‘typical patient’ rather than the percentage of their patients for whom this is a reason, but the rank order of reasons by patients and physicians can be compared. The Spearman rank order correlation was 0.86 and the top five reasons were the same for patients and physicians. The one major discrepancy was forgetting, which was more highly ranked by patients than physicians.

**Table 2:** Patient (*n* = 530) and physician (*n* = 964) reported reasons* for insulin omission/non-adherence†

Reason	Patients % and rank	Physicians % and rank

Too busy	18.9% 1	41.9% 3
Travelling	16.2% 2	43.6% 2
Skipped meal	15.0% 3	44.8% 1
Stress or emotional problems	11.7% 4	32.2% 5
Embarrassing to inject in public	9.7% 5	36.8% 4
Challenging to take it at the same time everyday	9.4% 6	29.1% 6
Forgot	7.4% 7	2.0% 11
Too many injections	6.0% 8	26.4% 7
Avoid weight gain	4.0% 9	13.4% 9
Regimen is too complicated	3.8% 10	16.8% 8
Injections are painful	2.6% 11	7.8% 10

* Respondents were asked to select top three reasons (order of reasons randomized, ‘Forgot’ responses volunteered as Other); data are % of respondents choosing a reason as one of the three.

† Absolute percentages reported for physicians cannot be compared with percentage of patients who report these reasons because physicians report whether this is a reason for the behaviour of their ‘typical patient’ rather than the percentage of their patients for whom this is a reason; rank order of reasons by patients and physicians can be compared.

Patients and physicians reported a number of negative perceptions about insulin treatment (Table 3). The two most commonly reported difficulties patients have with insulin treatment (as reported by both patients and physicians) were the number of injections taken and taking insulin at prescribed times; these two aspects of insulin therapy were also among those receiving the highest level of dissatisfaction (as reported by both patients and physicians). Patients reported difficulty in adjusting insulin doses, in agreement with physician views of their success in this area. In general, physicians were more dissatisfied with insulin therapy than patients, with the exception of ability to control blood glucose where they were similar. However, blood glucose control was ranked second in patient dissatisfaction, but sixth in physician dissatisfaction. Finally, a majority of both patients and physicians felt that diabetes is restrictive and controlled patients’ lives and about half felt that it is hard to live a normal life while managing diabetes. Patient and physician agreement was strongest for the wishes that insulin should be flexible to fit patients’ lives and that good control with insulin should not require injections every day.

**Table 3:** Patient and physician perceptions of insulin treatment

Categories and items	Patients (*n* = 1530)	Physicians (*n* = 1250)

Patient difficulties*		
Taking insulin at prescribed time or with meals every day	27.6%*^1^	54.5%
Number of daily injections	23.1%	58.5%*^2^
Following healthcare professional instructions	16.9%	45.4%
Preparing injections	10.3%	35.0%
Adjusting insulin doses	16.8%	NA
Changing timing of insulin to meet daily needs	NA	57.7%
Dissatisfaction†		
Choose frequency of injections	17.6%	43.3%†^2^
Choose time of injections	15.2%	32.2%†^1^
Blood glucose control	15.8%	15.9%
Simplicity of regimen	12.9%	27.8%
Safety regarding low blood sugar	11.4%	32.0%
Insulin treatment overall	10.0%	18.2%
Opinions‡		
Wish for good control with insulin not injected every day	92.5%	91.2%
Wish insulin regimen would fit daily life changes	81.4%	85.8%‡^2^
Insulin-treated diabetes controls life	66.7%	66.1%‡^1^
Insulin regimen can be restrictive	59.8%	68.2%
Hard to live normal life while managing diabetes	54.4%	49.6%

* Very difficult or somewhat difficult (vs. very easy, somewhat easy, not applicable, ‘don’t know’). Absolute percentages reported for physicians cannot be compared with percentage of patients for whom this is difficult because physicians report whether this is difficult for their ‘typical patient’ rather than the percentage of their patients for whom this is difficult; rank order of reasons by patients and physicians can be compared.

* ^1^Average of response for two items (insulin at prescribed times, insulin with each meal).

* ^2^Physician item is ‘taking insulin frequently’.

† Very dissatisfied or somewhat dissatisfied (vs. very satisfied, somewhat satisfied, not applicable).

† ^1^Physician item is ‘insulin regimens that better fit patients’ dynamic lives’.

† ^2^Physician item is ‘total number of injections per week’.

‡ Strongly agree or somewhat agree (vs. strongly disagree, somewhat disagree, neither, ‘don’t know’).

‡ ^1^Physician item is if his/her patients feel diabetes controls their lives.

‡ ^2^Physician item is ‘which insulin treatments could be more flexible’.

NA, not asked.

Table 4 examines country differences for two key measures reported by both patients and physicians—assessment of insulin omission/non-adherence and overall dissatisfaction with insulin treatment. Patient, specialist physician and primary care physician responses differed significantly (*P* < 0.05) across countries for all measures. Specialists reported significantly (*P* < 0.05) more insulin omission/non-adherence than primary care physicians, both overall and within most countries, but did not differ in dissatisfaction with insulin treatment (physician responses are not comparable with patient responses). Patients were significantly (*P* < 0.05) less satisfied with insulin treatment than either specialist physicians or primary care physicians, both overall and within most countries. Country rankings of dissatisfaction by patients, specialists and primary care physicians were not significantly correlated (Spearman’s rho < 0.5); country rankings of omission/non-adherence were stronger (Spearman’s rho > 0.5), although only specialist and primary care physicians rankings were significantly correlated, indicating that there may be a country effect for insulin omission/non-adherence.

**Table 4:** Comparison of patient and physician belief and behaviour by country

	Non-adherence**						Dissatisfaction					
		
			Physician*						Physician*			
						
Country	All patients*†% and rank		Specialist*% and rank		Primary care % and rank		All patients*†% and rank		Specialist*% and rank		Primary care % and rank	

China	33.3%	5	80.0%	4	68.0%	4	7.3%§¶	5	28.0%	2	18.0%	4
France	19.4%	8	70.0%	6	52.0%‡	7	3.1%§¶	7	16.0%	6	18.0%	5
Germany	39.8%	4	96.0%	1	86.0%‡	2	2.1%§¶	8	8.0%	7	26.0%‡	2
Japan	43.8%	1	74.0%	5	58.0%‡	6	21.5%¶	2	32.0%	1	36.0%	1
Spain	22.5%	7	56.0%	7	40.0%‡	8	9.9%	3	12.0%	3	14.0%	6
Turkey	24.1%	6	58.0%	8	64.0%	5	22.5%§¶	1	6.0%	8	10.0%	8
UK	41.4%	3	96.0%	1	78.0%‡	3	4.7%§¶	6	20.0%	5	22.0%	3
USA	41.9%	2	90.0%	3	94.0%	1	8.4%	4	12.0%	4	12.0%	7
Total	33.2%		77.5%		67.5%‡		10.0%§¶		17.0%		19.5%	

* Responses in this study populations differ significantly by country (*P* < 0.05).

† No significant differences between patients with Type 1 diabetes and those with Type 2 diabetes.

‡ Responses for specialist and primary care physicians differ significantly (*P* < 0.05).

§ Responses for patients and specialist physicians differ significantly (*P* < 0.05).

¶ Responses for patients and primary care physicians differ significantly (*P* < 0.05).

** For non-adherence, absolute percentages reported for physicians cannot be compared with percentage of patients who report insulin non-adherence because physicians report whether ‘any of your typical patients fail to take their insulin as prescribed’ rather than the percentage of their patients who have missed their dose or fail to take their insulin exactly as prescribed; rank order of countries for patients and physicians can be compared.

**FIGURE 2:**
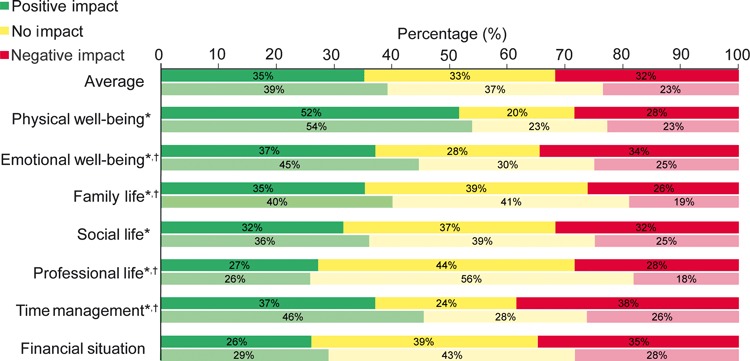
Patient reports: impact of insulin treatment on life domains in patients with Type 1 (non-hatched) and Type 2 (hatched) diabetes mellitus (*n* = 1530). *Positive and negative responses differ significantly (*P* < 0.05); †reports from patients with Type 1 and Type 2 diabetes differ significantly (*P* < 0.05).

In spite of the negative attitudes and perceptions of insulin treatment, Fig. 2 shows that more patients reported positive than negative impact on life for all domains except finances (*P* < 0.05), although the trend was stronger for patients with Type 1 diabetes than for those with Type 2 diabetes.

**FIGURE 3:**
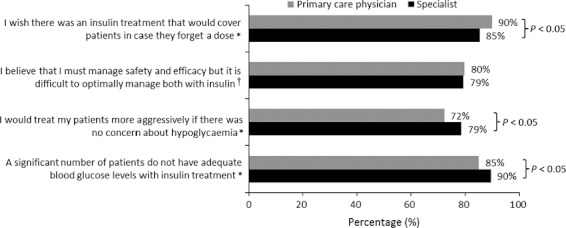
Physician beliefs about insulin treatment (*n* = 1250). *Strongly agree or somewhat agree (vs. strongly disagree, somewhat disagree, neither, ‘don’t know’); †strongly agree or somewhat agree (vs. strongly disagree, somewhat disagree)

Figure 3 presents physician beliefs about insulin treatment. While there are statistically significant differences between specialist and primary care physicians, physicians generally agree that many patients on insulin are not adequately controlled. Physicians report that the possibility of hypoglycaemia limits treatment aggressiveness, and that it is difficult to manage efficacy (hyperglycaemia) and safety (hypoglycaemia) simultaneously. Finally, physicians wish there was an insulin treatment that would have sustained efficacy if patients miss a dose or, to extrapolate, delay a dose.

## Discussion

Physicians perceived patients as relatively unsuccessful in terms of starting insulin when needed, adjusting insulin doses and adhering to prescribed regimens. Insulin omission/non-adherence was a common problem in all countries, although the rate was twice as high in some countries as in others. Physicians were aware of this problem, especially specialists, and reported that it was more common for mealtime and premixed insulin than for basal insulin. Physicians and patients generally agreed on the ranking of reasons for insulin omission/non-adherence; in addition to situationally appropriate reasons (e.g. after skipping a meal), respondents identified logistical problems (too busy, travelling) and psychosocial problems (stress/emotions and embarrassment) as key factors. Patients also volunteered that forgetting was a common reason, a possibility that was not as salient for physicians.

Several negative perceptions of insulin treatment were reported. Taking insulin at the prescribed time and frequency were the difficulties identified as most common by both patients and physicians. A majority of patients and physicians agreed that insulin regimens were restrictive and can control one’s life. Patient and physician dissatisfaction with insulin treatment varied substantially across countries, with no strong evidence of a consistent ranking of patient and physician levels of dissatisfaction across countries. Patient dissatisfaction with various aspects of insulin treatment was low (10–20%). Physician dissatisfaction with insulin treatment overall and with glucose control was in the same range as patients, but more physicians (25–45%) were dissatisfied with several other aspects of insulin treatment, including regimen simplicity, hypoglycaemia and injection timing and frequency.

Respondents also expressed opinions about how insulin treatment could be improved. Patients and physicians reported that they would like insulin treatment to be more flexible so that it could be adapted to situational variation in daily activities. Both groups indicated that it would be better if insulin did not have to be injected everyday, and physicians indicated that it would be an improvement if insulin would maintain its efficacy when patients miss a dose. Physicians also indicated that they would be more aggressive in treating diabetes if there was no concern about hypoglycaemia, suggesting that insulins with less risk of hypoglycaemia could be used more aggressively, potentially leading to improvements in blood glucose control and reductions in complications that result from suboptimal glucose control.

In spite of the drawbacks of current insulin regimens, patients reported that the net impact of insulin on their lives was positive. In six of seven domains examined (the exception was financial impact) more patients reported the impact of insulin to be positive than to be negative, with a substantial number saying it had no impact. The advantage was most pronounced in terms of physical well-being, but also was present for emotional well-being, as well as social relationships and work. The advantages were more pronounced in patients with Type 2 diabetes, especially in areas other than physical well-being. However, the other data presented here identified a number of unmet needs that could be addressed by adjusting regimens for currently available insulins and/or developing improved insulins.

There were substantial differences among the countries in respondents’ insulin-related beliefs and behaviours. However, the pattern of country differences was not consistent across measures or subgroups of respondents. Thus, the explanation for the country differences must be more complex than a set of country-specific clusters of insulin-related beliefs and behaviours that are shared by all members of a culture. This suggests that country differences reflect the interplay of cultural beliefs, healthcare provider training and health system characteristics.

### Research implications

Insulin omission/non-adherence is common and further research is needed to determine the risk factors associated with its occurrence. Additional attention should also be given to the problems of initiating insulin therapy and getting patients to self-manage their insulin doses. Research is needed to better understand physician concern about hypoglycaemia, especially as it affects their choices of diabetes management goals and strategies. Research in these areas could help to identify measures to improve patient and physician diabetes management and outcomes.

### Clinical implications

Although patients using insulin are not entirely satisfied with their treatment, insulin is well received. The primary problem is that it is seen as restrictive, making it difficult to take all doses as prescribed, especially given patients’ difficulty in adjusting insulin doses to respond to daily changes. Physicians should consider prescribing more flexible insulin regimens and reducing the burden of the treatment regimen. An ideal regimen would minimize the number of injections required [21], the risk of hypoglycaemia and the consequences of a delayed or missed insulin dose.

## Competing interests

MP has received research grant support from: Amylin, Animas, Genentech, MannKind, Medtronic MiniMed and Novo Nordisk. He has received consulting fees from: Amylin, Animas, Eli Lilly, Genentech, MannKind, Medtronic MiniMed and Novo Nordisk. He has received speaking honoraria from Novo Nordisk and has participated in scientific advisory committees for Eli Lilly, Novo Nordisk and Roche. He has ben reimbursed by Amylin, Animas, Eli Lilly, Genentech, MannKind, Medtronic MiniMed and Novo Nordisk for attending conferences.

AHB has received honoraria for lectures and advisory work as well as research funding from BMS/Astrazeneca, Boehringer-Ingelheim, Eli Lilly, Novo Nordisk, MSD, Roche Diagnostics, Sanofi-Aventis and Takeda.

LFM has received research grant support from Biodel, MannKind, Medtronic, Novo Nordisk, Pfizer and Sanofi-Aventis. He has received consulting fees from Biodel, Nipro and Novo Nordisk. He has received speaking honoraria from Amylin, Eli Lilly, Merk, Novo Nordisk and Sanofi-Aventis.

PMSD has received funds for research support, consulting, speaking, organizing education and staff support from Astrazenica, Berlin Chemie, BMS, Boehringer-Ingelheim, Eli Lilly, GSK, MSD, Novartis, Novo Nordisk and Sanofi-Aventis.
